# Epigenetics and environment in breast cancer: New paradigms for anti-cancer therapies

**DOI:** 10.3389/fonc.2022.971288

**Published:** 2022-09-15

**Authors:** Chitra Thakur, Yiran Qiu, Yao Fu, Zhuoyue Bi, Wenxuan Zhang, Haoyan Ji, Fei Chen

**Affiliations:** ^1^ Department of Pathology, Stony Brook Cancer Center, Stony Brook, NY, United States; ^2^ Department of Pathology, Renaissance School of Medicine, Stony Brook University, Stony Brook, NY, United States

**Keywords:** breast cancer, epigenetics, DNA methylation, chromatin modification, metabolism, environment, therapies

## Abstract

Breast cancer remains the most frequently diagnosed cancer in women worldwide. Delayed presentation of the disease, late stage at diagnosis, limited therapeutic options, metastasis, and relapse are the major factors contributing to breast cancer mortality. The development and progression of breast cancer is a complex and multi-step process that incorporates an accumulation of several genetic and epigenetic alterations. External environmental factors and internal cellular microenvironmental cues influence the occurrence of these alterations that drives tumorigenesis. Here, we discuss state-of-the-art information on the epigenetics of breast cancer and how environmental risk factors orchestrate major epigenetic events, emphasizing the necessity for a multidisciplinary approach toward a better understanding of the gene-environment interactions implicated in breast cancer. Since epigenetic modifications are reversible and are susceptible to extrinsic and intrinsic stimuli, they offer potential avenues that can be targeted for designing robust breast cancer therapies.

## Breast cancer overview

Cancers of the breast are the most prevalent malignancy observed in women worldwide. In the year 2022 alone, it is estimated that in the United States, nearly 287,850 new cases of invasive breast cancer and 51,400 new cases of ductal carcinoma *in situ* (DCIS) would be diagnosed, while 43,250 breast cancer deaths would occur (1). Breast cancers if diagnosed at an early stage, can significantly enhance the effective treatment strategies and improve the survival. The five-year survival rate for early detection is more than 90%, whereas it is reduced to 25% for patients diagnosed at the advanced stages (2).

**Figure 1:**
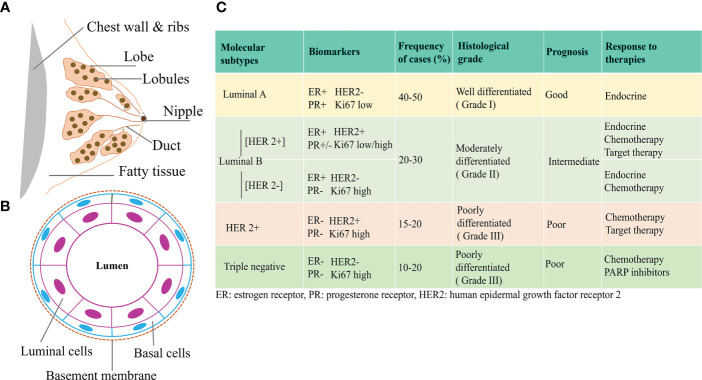
Classification of Breast Cancer **(A)** Breast showing the different tissue types consisting of duct, lobe, lobules, nipples, and fatty tissue. **(B)** Cross-sectional view of mammary duct, consisting of basal cells and luminal cells. Breast cancer arising from the luminal or basal cells can be further characterized based on the expression of different hormone receptors. **(C)** Based on the expression of ER, PR, HER2, and proliferation status as assessed by Ki67, different molecular subtypes of breast cancer have been identified that have distinct prognostic features and response to therapies (3).

Breast cancer is a highly heterogeneous disease and research is still ongoing to clearly understand its origin and the underlying mechanisms. The breast consists of milk producing glands and the connective tissues comprising the fibrous and fatty tissues. Lobules are the milk producing glands, and ducts carry the milk to the nipples, **Figures 1A, B**. Most breast cancers begin in the ducts or the lobules and based on the metastatic spread, they can either be benign or invasive. Ductal carcinoma *in situ* (DCIS) is considered as non-invasive and early-stage breast cancer confined to the milk ducts. If cancer originates in the ducts or lobules and metastasizes, they are considered invasive ductal carcinoma (IDC) and invasive lobular carcinoma (ILC) respectively. Almost, 80% of breast cancers belong to the IDC category (4, 5).

With the emergence of new high-throughput technologies and gene expression profiling, breast cancer has been molecularly characterized into distinct subtypes based on the expression of hormone receptors and proliferation statuses. Activation of human epidermal growth factor receptor 2 (HER2), estrogen receptor (ER), progesterone receptor (PR), proliferation marker Ki67, and/or mutations in the Breast Cancer (BRCA) gene, has been utilized in the histological and molecular characterization of breast cancer. These molecular subtypes are clinically divided into major forms that include Luminal A, Luminal B, HER2-enriched, and basal/triple negative breast cancer (TNBC). Luminal A cancer can either be ER and/or PR positive (+) or HER2 negative (-). Luminal B tumor can either be ER+ and/or PR+ or PR- and/or HER2+/-. HER2 overexpressed tumors constitute the HER2 enriched group, while TNBC lacks the ER, PR, and HER2 statuses. Luminal A tumors have low Ki 67 levels, are of low grade, and have the best prognosis, compared to Luminal B which have high Ki 67 levels and are usually high grade. Among all, TNBCs, have the worst prognosis and are aggressive due to high metastatic behavior (6–8). Such an existence of multiple subtypes of breast cancer is associated with distinct clinical behaviors/responses and has significant implications in breast cancer therapies (9, 10), **Figure 1C**.

Genetic predisposition or family history constitutes almost 10% of all breast cancer cases. Mutations in the BRCA gene, *BRCA1* and *BRCA2* is the most common germline aberrations associated with breast cancer having a collective 70% lifetime risk of developing breast cancer (11, 12). In fact, 15 to 20% of all TNBC cases are linked with the germline mutations in *BRCA1* or *BRCA*2 *(* 13) and in US, 12% of breast cancers are contributed by TNBCs with a 5 year survival rate of 8 to 16 percent only (14). Studying a series of early breast cancers revealed that the most frequently amplified genes in the tumors are the *p53, Myc, PTEN, PIK3CA, ERBB2, CCND1, GATA 3* and *FGFR1 (* 15). The risk of developing breast cancer is high in patients harboring mutations in the *BRCA1*, *BRCA2*, *TP53*, and *PTEN* genes (16). In addition to the genetic factors, breast cancer microenvironment plays a major role in its development and progression where the immune cell repertoire is cardinal (17).

Heightened or prolonged exposure to estrogen contributes to the major risk factor for breast cancer development. The occurrence of sporadic breast cancers is associated with exposure to estrogen, which is a substantial risk factor for the development of such cancers (18). Other risk factors include old age, obesity, high breast density, alcohol intake, smoking, hormonal therapy, and pregnancy associated factors (19–24). Additionally, early menarche/late menopause, usage of oral contraceptives, hormone replacement therapy, benign lesions, and radiation therapy are some of the known risk factors (25–28). Few of them are modifiable risk factors such as lifestyle and physical activity if adopted successfully, can offer reduction in the disease burden (29).

## Epigenetic players in breast cancer

Dynamic and heritable modifications occurring to the genome independently of DNA sequence, is a phenomenon referred to as the “epigenetics”. Interestingly, cancer was the first disease linked to epigenetic changes (30). For the onset of cancer, the activation of oncogenes and/or the suppression of tumor suppressor genes are the key events that are always accompanied with epigenetic changes. These epigenetic changes include DNA methylation, histone posttranslational modifications, expression of micro-RNA, and long non-coding RNA (31, 32).

**Figure 2:**
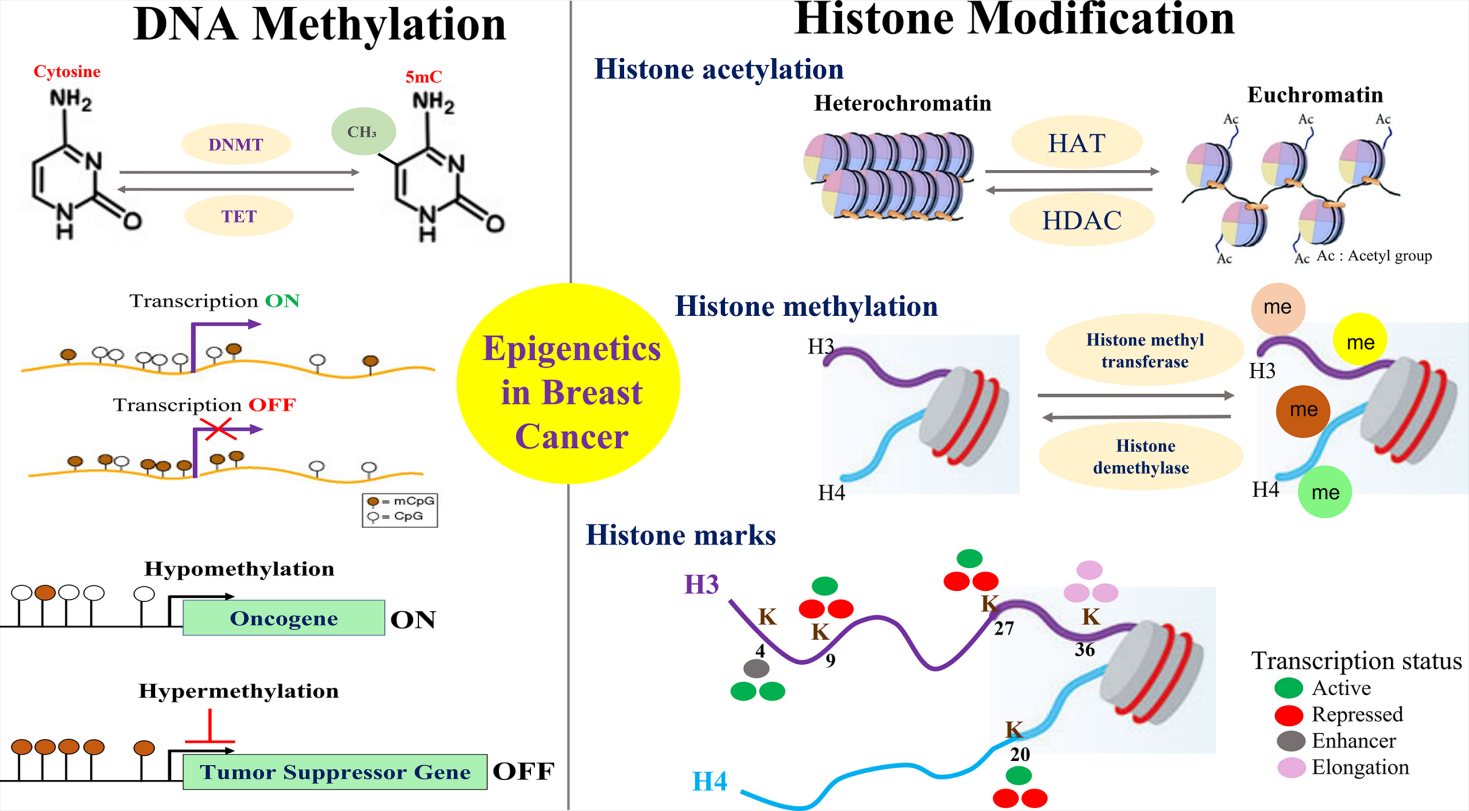
Overview of Key Epigenetic Events in Breast Cancer. Mechanisms for epigenetic alterations in breast cancer are shown focusing on two major players that include the methylation of DNA and the modification of histone proteins. Hypomethylation of oncogenes and hypermethylation of tumor suppressor genes is an important epigenetic phenomenon in breast cancer that affects various cellular processes of proliferation, apoptosis, migration, invasion, drug resistance, etc. Post translation modifications made to histone proteins impact gene expression by altering the chromatin structure towards open or closed conformation. Histone methylation of lysine is implicated in both transcriptional activation and repression depending on the methylation site that constitutes the various histone marks/code.

Breast cancer development is a complex and multistep process involving the synergistic crosstalk between genetic and epigenetic alterations which are influenced by a plethora of internal and external factors. Such factors include but not limited to the cell’s intrinsic microenvironment, nutrient supply, cellular stress as well as external environmental exposures to agents that are endocrine disrupters or are of carcinogenic nature. Altogether, critical genes involved in proliferation, apoptosis, cell motility, invasion, etc. are influenced by the epigenetic changes that are implicated in breast cancer development and progression (**Figure 2**).

### DNA methylation

One of the most well-known and major epigenetic mechanisms is DNA methylation, which involves the covalent addition of a methyl group (CH_3_) to the 5′-position of cytosine that resides before the guanine in the DNA sequence. Such methylation within the CpG dinucleotides which are concentrated in large clusters also called the CpG islands, regulates gene expression thereby governing the major biological process implicated in cancer (33, 34). As a result of methylation, a 5-methylcytosine (5mC) structure is formed that can either block the access of transcription factors to the binding sites of the DNA or engage methyl binding domain proteins (MBDs) in conjunction with the modification of histone proteins, so that the expression of methylated genes is prevented. In such a scenario when the promoters of key tumor suppressor genes are densely methylated, leads to their silencing and if oncogenes are less methylated, leads to their aberrant activation (35, 36).

DNA methylation is a reversible process where a specific group of enzymes called the DNA methyltransferase (DNMTs) govern the process. DNMT1, DNMT3a, and DNMT3b are the three active DNA methyltransferases. Demethylation of DNA is catalyzed by an enzyme family belonging to the Ten-eleven translocation methylcytosine dioxygenases also known as ten-eleven translocations (TETs), which can turn 5mC to 5-hydroxymethylcytosine (5-hmC) by the process of hydroxymethylation. TET1, TET2, and TET3 are three such enzymes involved in DNA demethylation thereby recovering the silenced genes that are once affected by the DNMTs. Together, this entire process influences the transcriptional activation of important genes involved in carcinogenesis and genomic stability (37–41). Several other proteins that have DNA demethylase activities and are implicated in breast cancer include the growth arrest and DNA-damage inducible protein (GADD45) and the cytidine deaminases family of proteins, Activation-induced cytidine deaminase (AID) and Apolipoprotein B mRNA editing catalytic polypeptide‐like family (APOBEC). GADD45A has compelling associations between DNA repair and epigenetic gene regulation (42, 43). In breast cancer, the interaction between GADD45 and BRCA1 gene has been suggested to influence the pathogenesis of the disease most likely *via* triggering the nucleotide excision repair mechanisms (44). Interestingly, GADD45A is abnormally methylated in breast cancer (45). AID proteins have important roles in the active DNA demethylation, where its engagement in the deamination of 5-mC to thymine has been reported (40, 46). Also, AID is known to facilitate DNA demethylation and is essential for the EMT in non-transformed mammary epithelial cells (47). Furthermore, while, APOBEC1 possesses DNA demethylase activity (48–50), APOBEC mutagenesis influencing the tumor evolution in ER+/HER2-breast cancer has been reported (51). Most recently it was shown that the APOBEC mutagenesis prohibited the growth of breast tumors by eliciting immunogenic responses (52).

Several genes in breast cancer exhibit CpG island hypermethylation (53) and in several instances, abnormal activity of DNA methyltransferases led to the hypermethylation and silencing of *HOXA5, TMS1, p16, RASSF1A*, and *BRCA1* genes of tumor suppressor behavior (54–56). Additionally, genes that are silenced due to promoter hypermethylation include *E-cadherin, TMS1*, *GSTP1*, and *p16 (* 57–59). These genes are involved in major biological processes such as estrogen signaling, pro-apoptosis (*HOXA5, TMS1*), cell cycle check points (*RASSF1A, p16*) and DNA repair mechanisms (*BRCA1*). While one of the best examples of a breast cancer susceptibility gene that is frequently silenced in sporadic breast tumors is the *BRCA1* gene, CpG hypermethylation of *BRCA1* associated with DNMT 3b overexpression has been reported (60). Early stages of sporadic breast cancer exhibit the loss of cell cycle checkpoint gene *p16INK4a via* aberrant CpG promoter methylation (61) and nearly 80% of breast tumors also exhibit a decreased expression of another cell cycle inhibitor gene *p21/CIP1* ^/WAF1^ via elevated methylation of *p21/CIP1* ^/WAF1^ gene (62).

DNA methylation also follows a distinct pattern that is displayed in different subtypes of breast cancer. For example, a high frequency of DNA methylation has been shown in ER+/luminal breast cancer compared to ER−/basal-like tumors (63, 64). Also, well-differentiated tumors have less methylated CpG islands in comparison to poorly differentiated breast tumors which exhibits a greater degree of methylated CpG islands (65). Similarly, increased promoter hypermethylation of the progesterone receptor gene has been observed in the PR negative breast tumors (66). Such a differential methylation pattern in the ER or PR or HER2 gene may affect the expression of these receptors on the breast tumor and hence can significantly impact the responsiveness of such tumors to relevant endocrine/hormonal therapies. In an attempt to study the DNA methylation profiles of the well-known expression subtypes of breast cancer i.e. luminal A, luminal B, and Basal like, 807 cancer associated genes were analyzed and it was revealed that there is variability in the methylation profiles of each of the three breast cancer subtypes and that the profiles are different from each other (64).

DNA methylation alterations in normal breast tissue or normal tissues adjacent to cancer can also give clues towards the likelihood of the occurrence of breast cancer. Interestingly, it is suggested that the detectable methylation variabilities in some of the cancer related genes in normal breast tissues can predate the occurrence of breast cancer (67). Moreover, distinct types of breast cancer can be tracked down back to the specific progenitor population, deploying their unique methylation profiles, thereby addressing the issues owing to their cell of origin or biological heterogeneity as observed in breast cancer (68). More recently, by comparing breast cancer to normal breast, seven breast cancer-specific methylation biomarkers have been identified, while six CpG sites are suggested to predict patient survival (69). Using a genome wide approach to analyze the DNA methylation and expression patterns in breast cancer and normal breast, *PRAC2*, *TDR10*, and *TMEM132C* genes have been identified that can serve as novel DNA methylation-gene markers of diagnostic and prognostic significance in breast cancer (70). Large scale integrative analysis of the DNA methylation profiles across 1538 METABRIC breast tumors with respect to transcriptional, genetic, and clinical aspects, revealed six global trends that affect the DNA methylation profiles of the breast. These trends consist of “contamination of immune and stromal cells”, “replication linked hypomethylation clock”, “X chromosome dosage compensation”, and “epigenetic instability at CpG islands”. Most importantly, this study identified X inactivation as a strong dosage compensation machinery, which can be the causative reason behind the methylation of attained X-associated loci in ER negative tumors (71).

### Chromatin modification

DNA is wrapped around histone proteins so that it can fit into the nucleus. Individual histone octamer consists of two copies of H2A/H2B dimer cores and H3/H4 tetramers, that wrap around 146 base pairs of the DNA. Nucleosomes comprise repeating histone units that ultimately make up the chromatin (72, 73). Histone octamer harbors an unstructured N terminal tail of differing lengths that protrudes outward from the nucleosome. This protruding amino terminal tail can be subjected to various kinds of modifications where chemical moieties are added. The addition of various chemical moieties or tags determines whether the DNA wrapped around histones is available for transcription. In case, when the chromatin is tightly folded, the DNA remains inaccessible to the transcription factors and hence the structure is transcriptionally silent, also called heterochromatin. Whereas when the structure is less condensed, more relaxed, and hence more accessible to the transcription factors and thereby remains transcriptionally active, also called euchromatin (74). There are at least four amino acid residues that are subjected to modifications, these include lysine, serine, tyrosine and arginine, and there are more than six kinds of modifications that can occur. These include methylation, acetylation, phosphorylation, ubiquitination, biotinylation, sumoylation, and proline isomerization. The different patterns of histone modifications, also famously referred as the histone code, influences the transition of the chromatin states between the euchromatin and heterochromatin eventually regulating gene expression (75, 76).

#### Histone acetylation

Post translational modifications made to histone proteins impact gene expression by altering the chromatin structure. Histone acetylation involves the addition of acetyl groups to the lysine residues of histones H3 and H4 by the group of enzymes known as the histone acetyltransferases (HATs) also called as “writers”. As a part of the gene regulatory machinery, such modifications disrupt histone-DNA interactions resulting in the unwinding of the nucleosome. HATs utilize acetyl CoA as a cofactor and catalyze the reaction, and in doing so they neutralize the positive charge on the lysine, thereby weakening the interaction between the histones and the negatively charged phosphate groups of the DNA. As a result, the condensed chromatin is now a more open and relaxed structure that is associated with a higher degree of gene transcription.

Acetylation is a dynamic and reversible process, where the acetyl groups can be removed by the group of enzymes called histone deacetylases (HDACs) also called “erasers”, resulting in the deacetylation of the histone lysine residues thereby making the chromatin more condensed and transcriptionally repressed (74, 76, 77). Acetylation of histone H3 on lysine 9 residue [H3K9], lysine 14 [H3K14], lysine 27 [H3K27], and lysine 122 [H3K122] has been associated with active transcription (78–80). It is interesting to note that DNA methyltransferases can directly interact with the HDACs and the methyl CpG binding domain family of proteins at their promoter regions and ultimately build a complex that is transcriptionally repressive. This repressive complex is critical for the conversion of acetylated histones that is transcriptionally active, to the deacetylated transcriptionally silent form (81).

Enzymes belonging to the category of histone acetylation “writers”, e.g., enzyme harboring the histone acetylation domains P300 is implicated in breast cancer where it is overexpressed and bestow towards an elevated risk of cancer occurrence and lower survival (82). P300/CBP, also modulate several processes associated with proliferation, cell death, epithelial mesenchymal transition (EMT), and metastasis in breast cancer (83–86).

There are important roles exerted by the histone deacetylases “erasers” where they regulate the cell growth, EMT, angiogenesis, and metastasis of breast cancer (87–95). For e.g., Sirtuins, a class III histone deacetylase family regulates the oncogenes and tumor suppressor genes thereby affecting the breast carcinogenesis in a dual fashion. In this context, SIRT1 hindered the TNBC tumorigenesis, whereas fostered the tumorigenesis of luminal subtypes (96, 97). Interestingly, SIRT1 functions downstream of the *BRCA1* gene and negatively regulate *Survivin*, an anti-apoptotic gene. Such transcriptional repression of *Survivin* is mediated *via* the deacetylation of histone H3 on lysine 9 on its promoter. Therefore, ablation of *BRCA1 via* lessened SIRT1 resulted in an upregulation of *Survivin* that facilitated the growth of breast tumors (98). Other Sirtuin family members are also implicated in breast cancer. For e.g., in TNBC cells, SIRT2 upregulation facilitated the deacetylation of histone H4 at the tumor suppressor gene *ARRDC3* and this rendered the aggressiveness of breast cancer (99). Also, SIRT7 is elevated in human breast cancers (100).

#### Histone methylation

Histone methylation mainly occurs on the side chains of lysine and arginine residues. Unlike acetylation, histone methylation does not alter the charge of the histone protein but involves the addition of the methyl groups. Depending upon the number of methyl moiety added, lysine can be mono, di, or tri methylated whereas arginine can be symmetrically or asymmetrically methylated (101, 102). A special group of enzymes called histone methyltransferases (KMTs) catalyze the transfer of a methyl group from the S-adenosylmethionine (SAM) to a lysine’s ϵ-amino group. Methylation is also a dynamic and reversible process where the removal of the methyl groups is carried out by demethylases (histone demethylases, KDMs). The consequences of histone methylation are more complicated and largely dependent upon the targeted residues. For example, methylation of lysine H3K4, H3K36, and H3K79 at histone H3 contributes to transcriptional activation, while methylation of lysine at H3K9, H3K27 on histone H3 and, H4K20 on histone H4 is associated with transcriptional repression and are considered repressive epigenetic marks (103). Some of the methylated lysine histone marks have a role in DNA repair e.g., H3K36me3 is important for the homologous recombinational repair of the DNA double strand breaks, and H4K20me3 aids the repair *via* non-homologous end joining process (104). The resulting balance between methyltransferases (also called “writer”) and demethylases (also referred to as “eraser”) determines the methylation status of the cell (105), where DNA methylation and histone acetylation act in coordination to govern the overall gene transcriptional regulation. The balance between the histone acetyltransferases (HATs “writer”) and histone deacetylases (HDACs “eraser”) control the overall chromatin states/structures, hence regulating the gene expression. Histone modifications offer novel targets that can be exploited in breast cancer therapies (106).

In breast cancer, luminal A subtypes are found to exhibit increased global acetylation and methylation of the histone protein in comparison to the basal subtype (107). By measuring the relative levels of seven modified histones proteins including H3K18ac, H3K9ac, H4R3me2, H3K4me2, H4K12ac, H4K16ac, and H4K20me3 in 880 invasive breast cancer patients, it was revealed that the expressions of all seven markers were negatively correlated with tumor grade. While the loss of H4K16ac was suggestive to be an early event in the pathogenesis of invasive breast cancer, reduced levels of H4R3me2, H3K9ac, and H4K16ac were significantly associated with large tumor size. High levels of H4R3me2 and H3K9ac correlated with low lymph node stage (107). Interestingly, the metastatic behavior of breast cancer was correlated to an increased H3K4 histone mark where the dynamics of H3K4 acetylation and methylation exemplify the different breast cancer subtypes. While breast cancer cells representing both early and late cancer cell phenotypes are associated with a genome-wide gain of H3K4ac; late-stage cancer cells exhibited a gain of H3K4me3 (108). PI3K/AKT signaling cascade plays a significant role in breast cancer progression and this signaling was found to regulate the methylation of H3K4 in breast cancer, where an elevated level of H3K4me3 was linked with breast tumors (109). Another histone mark, H3K27ac has an important role in breast cancer progression and is found to regulate the EMT process (110, 111). The loss of a repressive epigenetic mark, the H3K27me3 has been identified as a negative prognostic indicator in breast cancer (112). Strikingly, enrichment of H3K27me3 within the promoter of genes *FOXC1, RAD51, CDH1*, and *RUNX3*, resulted in enhanced cell growth and metastasis of breast cancer (113). Loss of Cadherin 1 due to its hypermethylation *via* DNA methylation and trimethylation of H3K27 has been reported during metastasis (114), where it is important to note that *Cadherin 1* is one of the key genes that inhibits metastasis and progression of breast cancer cells. Another mark, H4K20me3 is found to be significantly decreased in breast cancer and, importantly, it was an independent predictor of poor prognosis of the disease. This specific methylation of H4K20 is carried by the KMT5 family of enzymes that ultimately represses the transcription process (115, 116).

Among the enzymes implicated in gene regulation *via* epigenetic mechanisms, the enhancer of zeste homolog 2 (EZH2) is an important histone methyltransferase that methylates H3K27 leading to the transcriptional silencing of the target genes in breast cancer. Notably, in breast cancer, EZH2 has been found to be upregulated and promoted the EMT process (117, 118). Moreover, the level of EZH2 was gradually increased in breast cancer progression scenarios ranging from normal epithelium to epithelial hyperplasia, DCIS, IDC, and distant metastasis; and the expression of EZH2 was an independent predictor of breast cancer recurrence (119).

Members of the histone methyltransferases family, such as lysine methyltransferase 2 (KMT2) are also involved in the growth and spread of breast cancer cells, where they mediate the active histone methylation of H3K4 at the enhancer and the promoter regions of oncogenes and pro-metastatic genes, thereby facilitating the activation of genes that are estrogen dependent (120–123).

One of the only known histone 3 lysine 79 (H3K79) methyltransferases, is the histone methylase disruptor silencing 1 like (DOT1L) which has critical role in the development of breast cancer and is a potential therapeutic target for invasive breast cancer (124, 125). DOT1L is known to facilitate the aggressiveness of tumors by elevating the metastatic behavior of cancer cells (126) and is implicated in lymph node metastasis of breast cancer (127). In fact, targeting DOT1L by pharmacological interventions inhibited the growth and metastasis of TNBC cancer (128).

Among histone demethylases (erasers) family members are the prominent enzymes that are Fe^2+^/oxoglutarate-dependent containing a JumonjiC (JmjC) domain (129). Histone demethylase protein LSD1, a non JmjC demethylase has been found to negatively regulate the expression of cell growth and motility genes in breast cancer (130–133). Other JmjC KDMs involved in breast cancer are KDM4A, KDM4B and, KDM4C. Increased levels of KDM4A and KDM4B have been observed in ERα positive breast cancer cells, while TNBC cells showed an increased level of KDM4C (134). KDM4B regulates the cell cycle progression of breast cancer cells and is a direct target of ERα (135). While an increase of KDM3A is concomitant with a reduced H3K9me2/3 during breast tumorigenesis, KDM3A facilitated the activation of genes implicated in breast cancer as *MYC*, *PAX3*, *Cyclin D1*, *MMP-9*, *S100A4*, and *JUN*, thereby enhancing the proliferation and motility of breast cancer cells (136–138). KDM3A also promotes the growth of mammary gland ducts and tumors by positively affecting the proliferation *via* cyclin D1 (138). KDM4C is also necessary for breast cancer growth and, metastasis, where it serves as a co-activator of HIF-1α, with the underlying epigenetic mechanism of demethylating the H3K9me3 (139). Another histone demethylase PHF8 promoted EMT and breast tumorigenesis (140). PHF20L1, a methyl lysine reader protein containing a TUDOR domain, plays important role in breast cancer metastasis (141). Studies suggested its oncogenic role in response to hypoxic conditions, where it facilitated glycolysis, cell growth and metastasis of breast cancer cells by exerting its direct inhibitory activities on certain genes of tumor suppressive nature like *HIC1*, *KISS1*, and *BRCA1* (142).

### Non-coding RNAs

Functional RNA molecules that cannot be translated into proteins also referred to as non-coding RNA possess important regulatory effects and influence the expression of certain genes implicated in breast cancer. Among these are the long non-coding RNAs (lncRNAs) and micro-RNAs (miR). Micro-RNAs have been widely studied for its epigenetic regulation where they either activate or repress critical biological pathways and mechanisms important for breast tumorigenesis. Interestingly the let-7 family of micro RNAs has a significant role in breast cancer where its silencing has been associated with the development of metastasis and high-grade hormone negative breast tumors (143–145). Other micro-RNAs have important roles too. For. e.g., miR-9-3 activated apoptosis and miR-148a & miR-152 inhibited cell growth and angiogenesis (146, 147). Micro-RNAs involved in invasion and metastasis includes miR-125b, miR-126 and, miR-31 respectively (148–150). Some of the microRNAs whose aberrant hypermethylation has been reported in primary breast tumors include mir-663, mir-148, mir-9-1, mir-152, and mir-124a3 (151). Aberrant hypermethylation of H19, a lncRNA has been observed in invasive breast carcinoma when compared to normal breast tissues, where tumor suppressive functions of H19 have been suggested (152). HOTAIR, is another lncRNA where studies reported the recruitment of several writer proteins such as MLL1, MLL3, and P300/CBP to the HOTAIR’s promoter region thereby resulting in an enrichment of histone acetylation and elevation of H3K4me3, further driving the progression of breast cancer by suppressing the apoptosis (153).

**Figure 3:**
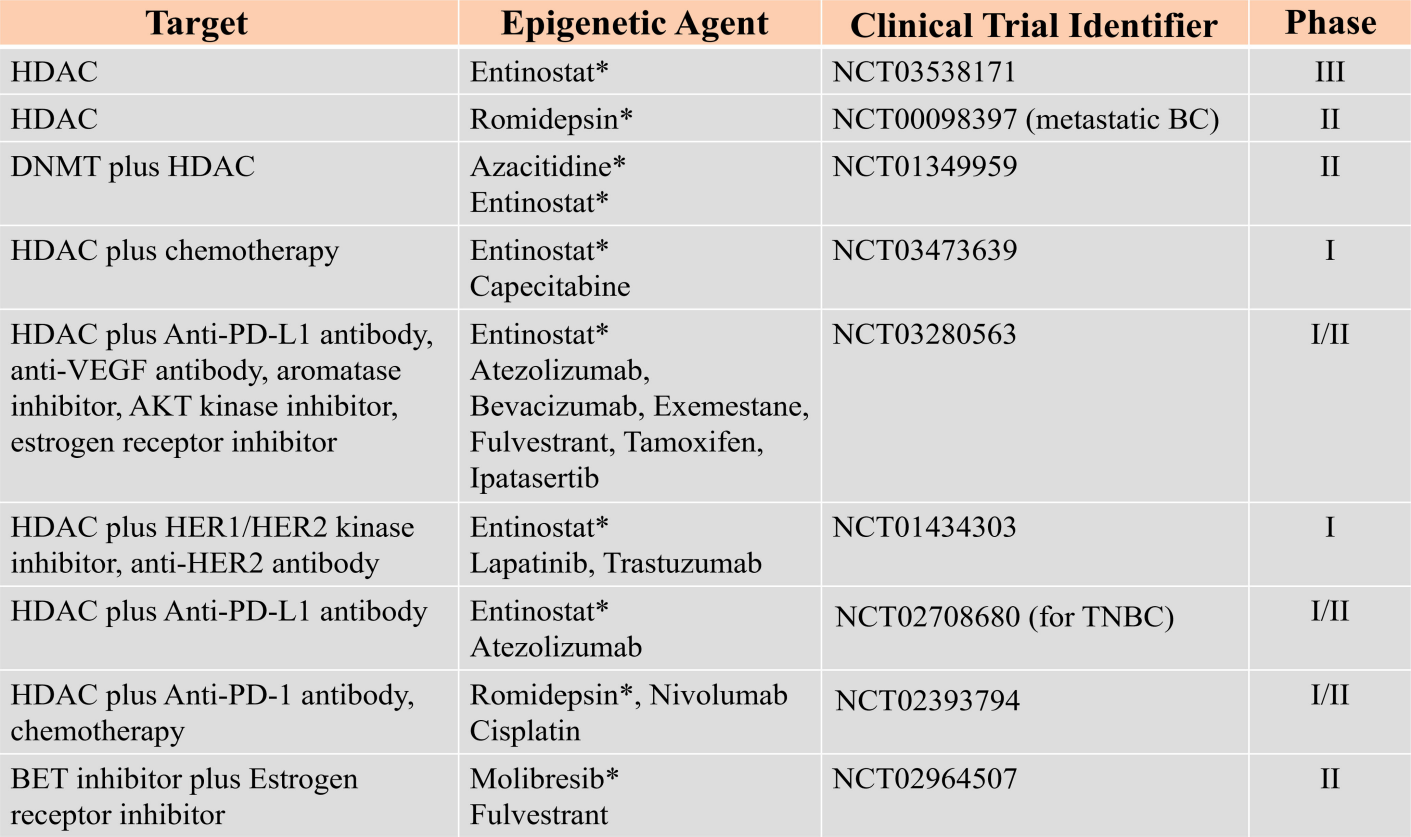
Epigenetic Targets and other combined inhibitors for breast cancer therapies under clinical trial. Data adapted from (154). Star (*) represents the specific epigenetic agent.

Therefore, epigenetic mechanisms offer many modalities that can be exploited for breast cancer therapies. Considering the fact that epigenetic changes induced by DNMTs and HDACs are transient and reversible, a number of studies are currently ongoing to establish effective, optimal dose and the treatment schedules for several epigenetic agents implicated in breast cancer, **Figure 3**. Data adapted from (154).

## Environmental triggers of epigenetic aberrations in breast cancer

In addition to family history and genetic predisposition, epidemiological studies unraveled the influence of environmental exposures to hormonal agents and other factors that can increase the risk for breast cancer development. Exposure to endocrine disrupters, indoor and outdoor air pollution, polycyclic aromatic hydrocarbons (PAHs) etc. can induce epigenetic changes in an exposure or disease relation fashion. Xenobiotics such as activators of the aryl hydrocarbon receptor (AHR), dioxin, phthalates, polychlorinated biphenyls (PCB), PAHs, bisphenol A (BPA), arsenic etc. prevalent in the environment, dietary items, soil, water, and other consumable products, are likely to contribute to the epigenetic dysregulation of oncogenes and tumor suppressor genes in breast cancer.

AHR is a well-known sensor and a regulator of toxic and carcinogenic responses to environmental insults (155, 156). In advanced malignant breast carcinomas, AHR is shown to be constitutively active (157) and several studies reveal that targeting AHR can offer a potential treatment option for breast cancer patients (158, 159). Industrial xenobiotics, dietary metabolites etc., serve as agonists of AHR and are ubiquitously present in the environment. AHR-mediated epigenetic repression has been found in the *BRCA1* gene which is also a direct target for AHR (160). In fact, CpG hypermethylation, deacetylation of H3K9, upregulation of H3K9me3, DNMT-1, DNMT-3a, DNMT-3b, and methyl-binding protein (MBD)-2 are some of the epigenetic changes linked with AHR mediated repression of *BRCA1* gene (161, 162).

BPA is yet another endocrine disrupter and is an epigenetically active xenoestrogen prevalent in plastic and food cans (163, 164) whose exposure has been linked with an increased risk of breast cancer (165). While overexpression of EZH2 is linked to breast cancer, *in-utero* exposure to BPA is able to alter the EZH2 expression in mammary tissues (166). In fact, exposure of normal breast cells to the environmentally relevant doses of BPA caused the ERα to internalize into the nucleus and also changed the DNA methylation status of a lysosomal associated membrane protein (LAMP3) (167). LAMP3 protein is implicated in metastasis and breast cancer cell motility and is of prognostic significance (168–170).

A very prevalent environmental contaminant of soil, food, and water is arsenic which has been studied widely for its carcinogenic effect. Exposure to arsenic and the risk of developing breast cancer has been reviewed extensively (171). Arsenic is able to transform the normal mammary epithelial cells that were subjected to chronic treatment with low levels. Moreover arsenic facilitated the growth of breast cancer cells that were ERα-positive (172, 173). The involvement of arsenic in the carcinogenesis process comes from the fact that it induces genomic instability mediated by disrupting the Fanconi anemia (FA) and/or breast cancer (BRCA) pathway (174). The epigenetic influence of arsenic has been established in studies reporting that arsenic influences DNA methylation by affecting the pool of available methyl groups. This is because the detoxification of arsenic utilizes methyl group from S-adenosyl-homocysteine (SAM) (175). Therefore, exposure to arsenic and its subsequent metabolism within the cells, impart towards a global hypomethylation owing to the usage of existing methyl stores available from SAM (176). Strikingly, maternal exposure to arsenic not only altered the DNA methylation but also increased the DNA methylation in children (177, 178).

The source of PAHs is myriad, which includes combustion products, automobile exhaust, cigarette smoke, indoor and outdoor air pollution, waste incinerators etc. (179). Tobacco smoking represents one of the important risk factors for breast cancer (180–182). Smoking not only affects the DNA methylation pattern of breast tumors, but it has been a critical factor linking DNA methylation and breast cancer for ER positive cancer subtypes (183, 184). Aberrant methylation alterations have also been observed in breast cancer cells exposed to benzo(*a*)pyrene, which resulted in the generation of DNA adducts at the CpG dinucleotides, ultimately affecting the epigenetic landscape of the methylation process (185).

External factors are not just limited to toxicants or environmental agents. The cellular microenvironment is sensitive to cues such as nutrient availability, hypoxia and, extracellular pH, and can epigenetically reprogram the metabolic behavior of cancer cells to adapt to the changing environment (186). The fact that metabolic profiles of cancer cells differ from the normal cells, gives us a clear indication of the underlying genetic and epigenetic machinery that are altered in the carcinogenesis process, thereby bestowing growth advantage to cancer cells for their survival. Hence metabolic reprogramming is indispensable for breast cancer and has many therapeutic ramifications (187). Cellular metabolites shuffling from the different cellular compartments such as cytoplasm, mitochondria, nucleus, etc., has the potential to regulate gene expression by altering the availability of enzymatic substrates and co-factors required for the metabolic reactions mediated epigenetic processes, such as DNA and histone modifications. Glucose remains one of the most important metabolites shaping the metabolic profiles of breast cancer by shifting the energy generating mechanisms from glycolysis to oxidative phosphorylation or vice versa. In this context, the availability of glucose affects the estrogen which facilitates glycolysis in a high glucose state but urged oxidative phosphorylation under the conditions of low glucose to meet the energy demands of the breast cancer cells (188). It is noteworthy that in adipose tissues, a major component of the breast, ERα is the vital regulator of a glucose transporter protein expression GLUT4 (189). Glycolysis can also be influenced by ERα, during the conditions of hypoxic stress. Hypoxia inducible factor-1α (HIF-1α) which is an oxygen-dependent transcriptional activator that carries out cellular adaptation to low oxygen and nutrient starved environment, is implicated in the ERα mediated activation of the glycolysis process in breast cancer (190). However, under normoxia and hypoxia conditions, both ERα and HIF-1α regulate histone demethylase JMJD2B and orchestrate breast cancer cell growth by epigenetically regulating the genes implicated in the cell cycle. Moreover, knocking down ERα can compromise the HIF-1α function even under hypoxic circumstances (135). One of the important transcription factors that aid cancer cells in metabolic adaption in a nutrient deprived environment, oxidative or xenobiotic stress is the nuclear factor erythroid 2-related factor 2 (NRF2) (191). Epigenetic modifications including DNA methylation are crucial for the regulation of NRF2 and its adaptor protein KEAP1 (192, 193). In breast cancer patients, elevated NRF2 expression led to decreased overall survival and disease-free survival (194). Elevated NRF2 enhanced the growth and motility of breast cancer cells by upregulating a pivotal enzyme of the pentose phosphate pathway, i.e., the glucose-6-phosphate dehydrogenase (G6PD) (195). In fact, Estradiol (E2) can stimulate NRF2 transcription, leading to an elevation in mitochondrial biogenesis (196).

## Mdig, an environment regulated gene in breast cancer

To ascertain the kind of risks and exposures affecting breast carcinogenesis, it is essential to gain an understanding of gene-environment interaction and the genes that are induced and manifested in breast cancer. Since a fraction of breast cancer cases is also sporadic, studying the genetic and epigenetic mechanisms that regulates breast tumor development under environmental and occupational settings, will undoubtedly offer new targets for chemoprevention and therapies.

We have identified one such environmentally induced gene named the Mineral dust-induced gene (mdig), also called MINA53, RIOX2, or NO52. Certain environmental agents such as mineral dust, tobacco smoke, arsenic, silica, etc. induced the expression of mdig (197–200). Mdig has oncogenic and epigenetic roles in a variety of human cancers, where it exhibits elevated expression (201, 202). Mdig promoted cell proliferation, cell cycle transition, and anti-apoptotic behaviors in different cell types, further corroborating its oncogenic role (198, 203). Mdig played key roles in the pathogenesis of arsenic induced lung cancer, where JNK-STAT3 signaling and mi-RNA21 mediate the processes. Further, we found that arsenic exposure induces the phosphorylation of EZH2 at serine 21 *via* JNK- and STAT3-dependent Akt activation (199, 204). Mdig is also upregulated in smokers in a pack-year dependent fashion, where it predicted poor overall survival in smokers that had lung cancer (205).

More recently, our studies on mdig and environmental factor arsenic revealed crosstalk between mdig and a master regulator of oxidative stress, NRF2, where together they contribute to arsenic induced generation of cancer stem like cells. Normal lung cells treated with arsenic showed an enhancement of HIF1α in the promoter of mdig, which was somehow accredited by activated NRF2 in response to arsenic (206). Since HIF1α is a direct transcriptional target of NRF2 (206) and considering the important role of NRF2 and HIF1 in tumorigenesis, our research further potentiates the importance of mdig on regulating the stress response activities implicated in genomic instability relying on metabolic reprogramming and cancer stem cells (207).

In breast cancer, we have identified that the expression level of mdig predicts the survival outcomes depending upon the different status of lymph node metastasis. A higher level of mdig predicted poor overall survival of patients who had no lymph node metastasis, whereas, in those patients who were positive for lymph node metastasis, high mdig expression predicted better overall survival (208). Dwelling further to assess the role of mdig in breast cancer, our studies revealed a negative correlation of mdig on the migration, invasion, and DNA methylation of breast cancer cells. Mdig not only regulated the chromatin accessibility of the migration/invasion genes but also exhibited a context dependent expression, where its expression was downregulated in invasive and triple negative breast cancer. This supported the notion that mdig is inhibitory for cell motility and spread and that’s why its high expression predicts favorable outcomes in lymph node metastasis positive cases of breast cancer (209). Since mdig is transcriptionally governed by an upstream regulator c-myc (210), which has both tumor accelerator and suppressive roles and can inhibit cancer metastasis (211), our studies are suggestive of the dual roles of mdig in breast cancer, where it is essential for the early stages of cancer development due to its pro-proliferative feature but is inhibitory in the later stages owing to its metastasis inhibitory features.

Mdig protein contains a conserved JmjC domain. Since JmjC domain has been identified as a signature motif of the JmjC family of histone demethylases (129), mdig’s involvement in the epigenetic process of histone modifications is inevitable. Recent studies provide evidence that the oncogenic activity of mdig is presumably achieved *via* its regulation on the demethylation of histone proteins. Our studies showed a demethylase like activity of mdig towards the repressive histone methylation markers that include H3K9me3, H3K27me3, and H4K20me3. Using the CRISPR-Cas9 gene editing approach coupled with chromatin immunoprecipitation sequencing (ChIP) in human lung epithelial cell line BEAS 2B, lung cancer cell line A549, and breast cancer cell line MDA-MB-231, an antagonistic effect of mdig on repressive histone trimethylation marks were revealed where mdig favored the open conformation of chromatin and permitted active gene transcription. Knocking down mdig resulted in a pronounced enrichment of these repressive trimethylation markers on the genes that are implicated in cell growth, stemness, inflammation, and metastasis (212). With the loss of mdig, there also occurred an increase in the levels of the polycomb repressive complex (PRC2) proteins EZH2 and RBBP4. Strikingly, these proteins are known to catalyze H3K27me3, and our previous studies identified a direct protein-protein interaction between mdig and CBX3, CBX5, RBBP4, and RBBP7 proteins. While RBBP4 and RBBP7 are the regulatory subunits of the PRC2 complex, CBX3 and CBX5 can recognize and bind to H3K9me3 (213).

In breast cancer cells, loss of mdig also enhanced an epigenetic mark of transcription elongation H3K36me3, in addition to H4K20me3 and H3K9me3. In this view, H4K20me3 being a marker for closed chromatin status in the somatic and embryonic stem cells (214), it is suggested that an elevation of H4K20me3 can contribute to growth inhibitory activities in the somatic cells. This notion is further supported by our previous studies where reduced mdig resulted in a decline of the S phase cells (198). It is also indicated that mdig acts as DNA demethylase or indirectly controls DNA methylation *via* the Tet family of DNA methylases (202). Additionally, a negative correlation was also observed between mdig and H3K9me3 in cellular studies (209, 215, 216). One of the consequences of enriched histone repressive marks H3K9me3 and H3K27me3, is on the transcription of genes implicated in glycan metabolism. Mdig exerted a positive regulatory role on the glycosylation process by inhibiting the repressive histone methylation marks (217).

Altogether, our research on mdig provided a much-needed rationale to explore its activities in several aspects of inflammation, stemness, metabolism, cell growth, metastasis, and epigenetic reprogramming orchestrating the carcinogenesis machinery in breast cancer.

## Perspectives

Despite tremendous progress being made in breast cancer research, some challenges still prevail. Metabolic plasticity, epigenetic reprogramming, and altered receptor repertoire lead to the issues of drug resistance and treatment failure. It is yet not fully clear as to what are the remarkable mechanistic programs that are critical for the breast tumor to become metastatic. Although our understanding of the heterogeneity of breast cancers has improved that has led to the generation of novel anti-cancer therapies exploiting the hormone receptor status, epigenetic marks, and other biological machineries, yet, when it comes to the general population there has been very limited success owing to the individual differences among the patients. An efficient personalized therapy would offer rescue to some extent towards combating the setbacks originated due to the heterogeneity and plasticity issues as observed in breast cancer therapies under clinical settings.

Environmental exposure to risk factors for breast cancer require particular attention, where relevant biomarkers related to such exposure need to be identified. Epigenetic mechanisms particularly DNA and histone methylation are involved in the onset of carcinogenesis by modulating the expression of potent oncogenes and tumor suppressors. Thus, dissecting the epigenetic elements would widen our knowledge towards better understanding the causative factors as well as the different routes that cancer cells adopt to attain heterogeneity. Moreover, studying maternal, *in utero* or pre-conception exposures and unraveling an association between the agents exposed and the different epigenetic repertoires correlating with the disease outcome, will be a promising avenue to explore. Such a strategy would assist in adopting modifiable approaches that can have significant implications in reducing the risk factors as a part of chemoprevention tactics. This demands a multidisciplinary effort that would integrate genomics, proteomics, and metabolomics in examining the different epigenomic profiles and pattern that drive the breast carcinogenesis under the conditions of sporadic and environmental settings. In this context, research on environmentally modulated genes engaged in breast cancer such as mdig, is warranted.

## Author contributions

CT and FC conceived the idea and wrote the article. YQ, YF, ZB, WZ, and HJ participated in conducting systemic review of the literature. All authors contributed to the article and approved the submitted version.

## Funding

This work was supported by National Institutes of Health (NIH) grants R01 ES031822, R01 ES028335, R01 ES028263, and Research Start-up fund of the Stony Brook University to FC.

## Acknowledgments

We would like to thank all the authors and researchers whose work has been cited here.

## Conflict of interest

The authors declare that the research was conducted in the absence of any commercial or financial relationships that could be construed as a potential conflict of interest.

## Publisher’s note

All claims expressed in this article are solely those of the authors and do not necessarily represent those of their affiliated organizations, or those of the publisher, the editors and the reviewers. Any product that may be evaluated in this article, or claim that may be made by its manufacturer, is not guaranteed or endorsed by the publisher.

## Glossary

**Table:**

DCIS	Ductal carcinoma in situ
IDC	Invasive ductal carcinoma
ILC	Invasive lobular carcinoma
HER2	Human epidermal growth factor receptor 2
ER	Estrogen receptor
PR	Progesterone receptor
BRCA	Breast Cancer gene
TNBC	Basal/triple negative breast cancer
lncRNAs	Long non-coding RNAs
miR	Micro-RNAs
MBD	Methyl binding domain
5mC	5-methylcytosine
5-hmC	5-hydroxymethylcytosine
DNMT	DNA methyltransferase
TET	Ten-eleven translocations
HAT	Histone acetyltransferases
HDAC	Histone deacetylases
HKMT	Histone methyltransferases
KDM	Histone demethylases
SAM	Sadenosylmethionine
EMT	Epithelial mesenchymal transition
DOT1L	Histone methylase disruptor silencing 1 like
JmjC	JumonjiC
PAH	Polycyclic aromatic hydrocarbons
AHR	Aryl hydrocarbon receptor
PCB	Polychlorinated biphenyls
BPA	Bisphenol A
LAMP3	Lysosomal associated membrane protein
HIF-1α	Hypoxia inducible factor-1&alpha;
GLUT4	Glucose transporter protein expressiona
G6PD	Glucose-6-phosphate dehydrogenase
Mdig	Mineral dust-induced gene
NRF2	Nuclear factor erythroid 2-related factor 2
ChIP	Chromatin immunoprecipitation sequencing
PRC2	Polycomb repressive complex
GADD45	Growth arrest and DNA-damage inducible protein
AID	Activation-induced cytidine deaminase
APOBEC	Apolipoprotein B mRNA editing catalytic polypeptide-like family
